# Supplementary material for: Capsule-based federated reinforcement learning adaptive sliding mode for anomaly detection and control of floating wind turbines
Source: PLoS One. 2025 Dec 4;20(12):e0336410. doi: 10.1371/journal.pone.0336410 (PMC12677533; doi:10.1371/journal.pone.0336410)
Supplement: Attachment — Submitted filename: review-comments.pdf [file pone.0336410.s002.pdf]

PLOS ONE

Review comments

Capsule-Based Federated Reinforcement Learning Adaptive Sliding Mode for Anomaly Detection and Control of Floating Wind Turbines.

by Hadi M. KhalafAnsar, PhD, Jafar Keighobadi and Mohsen Shahhosseini, Msc

Manuscript No : PONE-D-25-42083

Dear authors and editors:

This article proposes an intelligent collaborative control framework that capsule network, federated learning, deep reinforcement learning, and sliding mode control to address the challenges faced by floating wind turbines in complex marine environments, such as strong disturbances, uncertainties, and communication limitations. The authors claimed this work will improve the reliability and efficiency of floating wind turbines in turbulent weather conditions (short-term) and erratic ocean reception (long-term).

The manuscript is generally well organised and composed. Nevertheless, several revisions are still required.

The detailed review comments are as below:

- 1. The first issue I proposed is: this article only simulates under moderate sea conditions and does not consider extreme sea conditions such as typhoons, turbulent winds, and giant waves.
- 2. I don't follow how the complexity of WT was included and realized by the toolbox in Matlab, without introducing the governing eqautions of its movements.
- 3. The numerical method developed in Matlab seems to have not been validated by other researches, such as experimental results. Addtionally, it has not been seen that any comparison with other prevailing or state-of-art methods.
- 4. Some sentences in this article are lengthy and grammatically incorrect. Please have colleagues or professional editors who are native English speakers polish the language and standardize the reference format.

Cheers,

PLOS ONE
