# Supplementary material for: Capsule-based federated reinforcement learning adaptive sliding mode for anomaly detection and control of floating wind turbines
Source: PLoS One. 2025 Dec 4;20(12):e0336410. doi: 10.1371/journal.pone.0336410 (PMC12677533; doi:10.1371/journal.pone.0336410)
Supplement: Attachment — Submitted filename: Plos_Review1.docx [file pone.0336410.s004.docx]

Dear editor-in-chief of *Plos One*

We value your time in conducting a peer review of the paper titled “Capsule-Based Federated Reinforcement Learning Adaptive Sliding Mode for Anomaly Detection and Control of Floating Wind Turbines”. All remarks from both reviewers and the editor have been fully addressed. We hope that the appropriate adjustments will meet the approval of the considerate reviewers and editor.

**Editor’s comments**

When submitting your revision, we need you to address these additional requirements.

1. Please ensure that your manuscript meets PLOS ONE's style requirements, including those for file naming. The PLOS ONE style templates can be found at <https://journals.plos.org/plosone/s/file?id=wjVg/PLOSOne_formatting_sample_main_body.pdf> and <https://journals.plos.org/plosone/s/file?id=ba62/PLOSOne_formatting_sample_title_authors_affiliations.pdf>

**Response:** *We appreciate the editor’s suggestion to improve our work. The new version is written according to journal policies.*

2. Please note that PLOS One has specific guidelines on code sharing for submissions in which author-generated code underpins the findings in the manuscript. In these cases, we expect all author-generated code to be made available without restrictions upon publication of the work. Please review our guidelines at <https://journals.plos.org/plosone/s/materials-and-software-sharing#loc-sharing-code> and ensure that your code is shared in a way that follows best practice and facilitates reproducibility and reuse.

**Response:** *We appreciate the editor’s comment to share data. All data underlying the results are included within the article and its Supplementary Information.*

3. Please provide a complete Data Availability Statement in the submission form, ensuring you include all necessary access information or a reason for why you are unable to make your data freely accessible. If your research concerns only data provided within your submission, please write "All data are in the manuscript and/or supporting information files" as your Data Availability Statement.

**Response:** *We appreciate the editor’s suggestion. The aforementioned sentence was exactly added to the manuscript on page 22.*

4. Please amend your authorship list in your manuscript file to include author [Hadi M. KhalafAnsar](mailto:H_Mohammadian96@ms.tabrizu.ac.ir).

**Response:** *We appreciate the editor’s remarks. The author’s scholarly name was edited as Hadi Mohammadian KhalafAnsar.*

5. Please amend the manuscript submission data (via Edit Submission) to include author Hadi Mohammadian KhalafAnsar.

**Response:** *We appreciate the editor’s concise point. The author’s scholarly name was modified as Hadi Mohammadian KhalafAnsar.*

6. We note that Figure(s) 1,2, 3, and 4 in your submission contain copyrighted images. All PLOS content is published under the Creative Commons Attribution License (CC BY 4.0), which means that the manuscript, images, and Supporting Information files will be freely available online, and any third party is permitted to access, download, copy, distribute, and use these materials in any way, even commercially, with proper attribution. For more information, see our copyright guidelines: <http://journals.plos.org/plosone/s/licenses-and-copyright>.

We require you to either (1) present written permission from the copyright holder to publish these figures specifically under the CC BY 4.0 license, or (2) remove the figures from your submission:

a. You may seek permission from the original copyright holder of Figure(s) 1, 3, and 4 to publish the content specifically under the CC BY 4.0 license.

We recommend that you contact the original copyright holder with the Content Permission Form (http://journals.plos.org/plosone/s/file?id=7c09/content-permission-form.pdf) and the following text:
“I request permission for the open-access journal PLOS ONE to publish XXX under the Creative Commons Attribution License (CCAL) CC BY 4.0 (http://creativecommons.org/licenses/by/4.0/). Please be aware that this license allows unrestricted use and distribution, even commercially, by third parties. Please reply and provide explicit written permission to publish XXX under a CC BY license and complete the attached form.”

Please upload the completed Content Permission Form or other proof of granted permissions as an "Other" file with your submission.

In the figure caption of the copyrighted figure, please include the following text: “Reprinted from [ref] under a CC BY license, with permission from [name of publisher], original copyright [original copyright year].”

b. If you are unable to obtain permission from the original copyright holder to publish these figures under the CC BY 4.0 license or if the copyright holder’s requirements are incompatible with the CC BY 4.0 license, please either i) remove the figure or ii) supply a replacement figure that complies with the CC BY 4.0 license. Please check copyright information on all replacement figures and update the figure caption with source information. If applicable, please specify in the figure caption text when a figure is similar but not identical to the original image and is therefore for illustrative purposes only.

**Response:** *We appreciate the Editor's emphasis regarding our compliance with the journal's guidance on intellectual property. In accordance with standard academic practice, our first step was to seek the formal permission for the figures from the published paper "Capsule Routing via Variational Bayes" (Ribeiro et al., AAAI, 2020) by contacting the corresponding author via email. Unfortunately, we couldn't receive a reply. Consequently, to avoid any potential copyright issues and to better tailor the illustrations to our work's narrative, we have undertaken substantial modifications:*

*Fig 1: The upper section has been re-conceptualized and is now adapted from a different figure in our paper to serve our explanatory purpose.*

*Fig 2: This has been entirely replaced with an original diagram representing our proprietary model, which more effectively clarifies the proposed methodology and it has been appropriately cited to the mentioned paper.*

*Fig 3: The core idea from the original figure has been effectively merged into the revised Fig 2, making a separate figure redundant.*

*Fig 4: This is an original creation, developed using SolidWorks, and is presented here for the first time.*

*We are confident that these revised figures are distinct, fulfill their illustrative purpose more effectively, and fully comply with copyright standards.*

7. If the reviewer comments include a recommendation to cite specific previously published works, please review and evaluate these publications to determine whether they are relevant and should be cited. There is no requirement to cite these works unless the editor has indicated otherwise.

Reviewers' comments:

Reviewer's Responses to Questions

**Comments to the Author**

1. Is the manuscript technically sound, and do the data support the conclusions?

The manuscript must describe a technically sound piece of scientific research with data that supports the conclusions. Experiments must have been conducted rigorously, with appropriate controls, replication, and sample sizes. The conclusions must be drawn appropriately based on the data presented.

Reviewer #1: No

Reviewer #2: Yes

Reviewer #3: Partly

2. Has the statistical analysis been performed appropriately and rigorously?

Reviewer #1: N/A

Reviewer #2: Yes

Reviewer #3: Yes

3. Have the authors made all data underlying the findings in their manuscript fully available?

The [PLOS Data policy](http://track.editorialmanager.com/CL0/http:%2F%2Fwww.plosone.org%2Fstatic%2Fpolicies.action%23sharing/1/010f01994b395c21-3668d0d7-1f7d-4621-b8f9-ea83aeb4533e-000000/weLz4ICoe0bi4iH_r_RpRhaCEopaVqKt6IpJLMfHwHE=228) requires authors to make all data underlying the findings described in their manuscript fully available without restriction, with rare exception (please refer to the Data Availability Statement in the manuscript PDF file). The data should be provided as part of the manuscript or its supporting information, or deposited to a public repository. For example, in addition to summary statistics, the data points behind means, medians and variance measures should be available. If there are restrictions on publicly sharing data—e.g. participant privacy or use of data from a third party—those must be specified.

Reviewer #1: Yes

Reviewer #2: Yes

Reviewer #3: Yes

4. Is the manuscript presented in an intelligible fashion and written in standard English?

PLOS ONE does not copyedit accepted manuscripts, so the language in submitted articles must be clear, correct, and unambiguous. Any typographical or grammatical errors should be corrected at revision, so please note any specific errors here.

Reviewer #1: No

Reviewer #2: Yes

Reviewer #3: Yes

5. Review Comments to the Author

Please use the space provided to explain your answers to the questions above. You may also include additional comments for the author, including concerns about dual publication, research ethics, or publication ethics. (Please upload your review as an attachment if it exceeds 20,000 characters)

With all due respect and gratitude to the esteemed reviewers for their time and thoughtful and constructive comments. The responses to each of the points raised are provided in detail below. We believe that with these corrections, the paper has reached the high standards of PLOS ONE.

Response to Reviewer #1

We thank the great reviewer for their valuable check on the paper and their clear tips. We have looked at all the points. We made the right fixes in the new write of the text to meet their needs well.

1. Please rewrite the paper according the PLOS journal standard. Write it in clear English and proper formatting. And then submit again.

**Response:** *Thank you for the key critic. We all agree on the need for clear and pro-active words. The current form of the paper has been fully checked and repaired by experienced English professionals. It's to make sure that paper is clear, grammatically correct, and follows the rules of advanced writing. Each part of writing, setup, and how to list references follows the PLOS rules. We believe that the new form is clear and easy to grasp in both terms and in practice.*

*Corrections applied the entire of the paper context especially Table formats, Citation list format on page 22.*

2. The methodology section is unclear and not presented well. Please rewrite.

**Response:** *Thank you for your detailed feedback. We have thoroughly reviewed and rewritten the methodology section (Section 2) to increase its clarity and coherence. In the new edition, this section is organized with clearer subheadings and each step of the process is described in detail:*

*•* ***Modeling of the online adaptive intelligent control:*** *In this section, the main system equations and the reference model are clearly defined.*

*•* ***DRL Neural Network:*** *The logic of combining Sliding Mode Control (SMC) with Deep Reinforcement Learning (DRL) and the aim of this work to achieve stability and reduce the chattering phenomenon is explained in detail. The architecture of the hybrid neural network that uses Capsule Network and Federated Learning is described in more detail and the role of each component (Actor-Critic) in estimating the disturbances is specified.*

*This new structure makes it easier for the reader to understand the complex process of controller design and clearly shows the logical connection between the different components.*

*The clarification for the proposed method carried out on page 3 and relevant subsections.*

3. The figures are very poor.

**Response:** *We acknowledge this criticism and thank you for your attention to the visual quality of the paper. All figures in the paper, especially the complex flowcharts in Figs. 1 and 2, have been redesigned using specialized software and at high resolution (at least 300 DPI). Labels, legends, and axis titles have been made larger and clearer for better readability. We are confident that the current quality of the figures is quite standard and suitable for printing.*

*Corrections applied on pages 5 and 7.*

4. Please write a confident conclusion.

**Response:** *Thank you for this valuable suggestion. The conclusion section (Section 4) has been rewritten to more firmly and clearly state the key achievements of the research. In the new version, the main innovations of this research have been emphasized:*

*• Providing a novel hybrid control framework that combines the robustness of SMC with the intelligence and adaptability of DRL.*

*• Using capsule networks for accurate and hierarchical disturbance detection, which is a novel approach in this field.*

*• Applying federated learning for distributed training and data privacy preservation across multiple wind turbines.*

*The new conclusion clearly explains the importance of these findings in increasing the sustainability and efficiency of floating wind turbines and their potential impact on the renewable energy industry.*

*Corrections applied on page 21.*

Response to reviewer #2

We are grateful to the reviewer for their congratulations and insightful detailed comments. These points helped us greatly in improving the quality of the paper.

1. Introduction 1st paragraph - where did you get this information? This paragraph could be removed.

**Response:** *Thank you for your suggestion. The information presented in the first paragraph of the Introduction, which discusses the environmental impacts of fossil fuels, is based on reliable sources [1-3], which are referenced at the end of the rewritten paragraph. According to your worthful viewpoint, we corrected and shortened this paragraph which would increase the motivation of readers over the importance of the transition to renewable energy. We see it seems a little general duplication, but for remembering the harmful effects of fusil fuels over air, water, soil and Global Warming, we request you be kind to admit not totally its removal.*

*Corrections applied on page 1.*

2. Introduction highlights could be placed at the end of the section, just before section 2.

**Response:** *Thank you for your constructive suggestion. This is an excellent suggestion to improve the narrative flow of the text. So, as you mentioned, the list of research innovations (items 1-4) has been displaced to the end of the Introduction section.*

*Corrections applied on page 2.*

3. If possible, try to save figures in some other format with higher resolution, specially figures 1 and 2.

**Response:** *We completely agree. As also answered to reviewer #1, Figs. 1 and 2 and other figures have been reproduced in vector format (at least 300 DPI) and high resolution to ensure their clarity in print and digital display and that all details are easily visible.*

*Corrections applied on pages 5 and 7.*

4. Table 1 - Instead of using the * to explain the variables, you can put that information in the paragraph before the table: "... makes the theoretical framework robust and reliable. In Table 1 ...".

**Response:** *This is a very good suggestion and increases the readability of the text. The explanation of the variables of the federated learning algorithm, as you mentioned, has been moved from the footnote to the main text before Table 1, so that the reader can easily be familiar with all the parameters before reading the table.*

*Corrections applied on page 7.*

5. During section 2.3 you make a lot of assumptions during your mathematical formulation, such as simplifications, lemmas, functions. It would be good if they come with some references.

**Response:** *Thank you for this detailed technical note. Assumptions and mathematical steps in Section 2.3, especially in Lyapunov stability analysis, are based on physical facts, and standard/well-established principles in control theory. To satisfy your suggestion, the definition of candidate Lyapunov function and above-mentioned assumptions are based on authoritative sources such as [9, 14, 19] in the reference list of the text. For greater clarity, a sentence has been added in the text to emphasize that these approaches are rooted in the well-known technical literature and are not novel.*

*Corrections applied on page 8.*

6. In section 3, you quote the NREL model but there is no reference, please add that.

**Response:** *Thank you for pointing it out. This was an oversight on our part. The original reference for the NREL 5 MW wind turbine model (Jonkman et al. report) has been added to Section 3, where the model is first referenced.*

*Corrections applied on page 10.*

7. In Figure 5, is there any comments on why your method is smoother than the NREL data?

**Response:** *Thank you for pointing it out. Our controller’s enhanced smooth trajectory is attributed to its capability in accurately and instantaneously estimating complex disturbances and responding intelligently to them. This high precision is achieved through our DRL agent and two key technologies:*

*Capsule Network: This model creates a deep and multi-dimensional understanding of wind and wave conditions, modeling the system’s state far more accurately than linear models.*

*Federated Learning: This framework, by leveraging the collective experience of all turbines in a wind farm, renders the estimation model highly robust and generalizable.*

*Consequently, the combination of this precise disturbance estimation and the guaranteed stability of the SMC yields a more stable and smoother performance compared to the linear PI controller.*

*Corrections applied on page 15.*

8. In Fig. 6, is it possible to plot the error that the 2 functions are tracking? Same goes for Fig 7.

**Response:** *Thanks for this precise suggestion. In fact, Fig 6 shows exactly the tracking error itself (e(t)=X(t)−X_m_(t)). The main goal of the controller is to converge this error to zero, which is clearly evident in this figure. Similarly, Fig 7 shows sliding surfaces whose convergence to zero means that the error converges to zero. To avoid any ambiguity, the title of Fig 6 has been revised to explicitly refer to “State Tracking Error”.*

*Corrections applied on page 15.*

9. For observation 8, wouldn't the log plot be a better option? Also, why only 5 s of simulation?

**Response:** *We would like to express our sincere gratitude to the reviewer for their detailed and constructive feedback.*

1. *Regarding the use of a logarithmic plot: The reviewer’s suggestion about using a logarithmic scale is highly valuable, and we have indeed employed this approach in our original analysis. The primary objective of our controller is to attenuate the resonance peaks at the natural frequencies of the structure. The most standard and effective scientific tool to display and quantify this phenomenon is frequency domain analysis using a Bode plot, which inherently utilizes a logarithmic scale to represent the amplitude (in decibels, dB). As shown in the text and Fig 9, our analysis demonstrates that the proposed controller, SMC-DRL, suppresses the resonance amplitude in all degrees of freedom by more than 100 dB. This logarithmic reduction is a highly accurate and powerful measure of the controller’s ability to decouple disturbance energy from structural modes. This frequency domain analysis provides much deeper insights than a simple logarithmic-scale time-error plot, as it directly targets the controller’s performance at critical frequencies.*
2. *Regarding the short simulation time frame (5 seconds): The short time frame of 5 seconds for the particular plot is solely depicted due to magnifying of transient tracking errors in detail and the initial convergence speed of the controller, as well. The comprehensive analysis and main validation of the controller performance over longer time periods and under varying conditions have been drafted in Fig 4. As explained in the paper text, we injected a frequency-sweep chirp signal into the system to evaluate the robustness and adaptability of the controller. The time response to this signal (bottom-left graph in Fig 9) clearly shows the controller’s performance over an extended time period and in the face of time-domain varying disturbances, proving its visible superiority over the PID controller. This comprehensive test confirms the controller’s capability to maintain stability and to reject disturbances under varied and long-term operating conditions.*

*Corrections applied on pages 20 and 21.*

Response to Reviewer #3

We thank the reviewer for their detailed summary of the paper’s objectives and their constructive feedback.

1. The first issue I proposed is: this article only simulates under moderate sea conditions and does not consider extreme sea conditions such as typhoons, turbulent winds, and giant waves.

**Response:** *We sincerely thank the esteemed reviewer for raising this highly important and precise point.*

*The primary objective of this paper is to present and validate a novel control framework for* ***operational conditions****—that is, the common and variable sea states during which a floating wind turbine spends the majority of its operational life generating power. A key consideration is that under the extreme conditions you rightly highlighted such as storms, the turbine transitions out of its power-generating operational mode and enters a* ***survival or parked mode*** *to prevent structural damage. In this mode, the primary control strategy shifts entirely from power optimization to complete system shutdown and structural protection. Therefore, evaluating the performance of our proposed controller—which is specifically designed for power production optimization—under conditions where the turbine is shut down and in survival mode is, from a technical standpoint, not applicable.*

*Analyzing system behavior under extreme conditions constitutes a separate and highly complex research domain, focusing largely on structural load analysis and survival strategies. Nevertheless, we fully recognize the importance of this issue. Inspired by your valuable insight, we have highlighted the investigation of system behavior during the* ***transition to extreme conditions*** *as a significant future research direction in the "Future Work" section of the manuscript.*

*Corrections applied on page 21.*

2. I don't follow how the complexity of WT was included and realized by the toolbox in MATLAB, without introducing the governing equations of its movements.

**Response:** *Thank you for this comment and apologize for the lack of clarity. In Section 3 (Numerical Results for FWT), the governing equations of the turbine motion are described in detail.*

*• Eq. (25) formulates the overall forces acting on the system.*

*• Eq. (27) specifies the torques due to these forces.*

*• Most importantly, on page 12, the linearized state-space model of the system, including the matrices A and B, is fully presented. This model is exactly used for controller design and simulation in MATLAB/Simulink software. This approach (using state space model) is a standard method in control engineering for implementation and simulation of complex dynamic systems.*

*Clarification parts were highlighted on page 11.*

3. The numerical method developed in MATLAB seems to have not been validated by other researches, such as experimental results. Additionally, it has not been seen that any comparison with other prevailing or state-of-art methods.

**Response:** *We appreciate the reviewer’s valuable feedback. In this response, we address the comments in two parts:*

*Validation: Comprehensive experimental validation of these systems is often impractical for academic research due to the substantial costs and implementation complexities involved. Therefore, we have utilized the standard and accepted approach in this field, which is simulation using the well-established and validated NREL 5 MW wind turbine model. As presented in Fig 5, we first compared the performance of our baseline model with the published NREL results to ensure the accuracy and fidelity of our simulation. The excellent agreement between the two confirms the validity of our modeling approach.*

*Comparison with other methods: A direct and thorough comparison is provided in the manuscript. In Figs. 6 and 7, the performance of our proposed SMC-DRL based controller is directly benchmarked against another advanced intelligent controller, namely the radial basis function neural network (RBFNN)-based controller. RBFNN is a widely adopted and state-of-the-art technique in nonlinear control. The results clearly demonstrate the superiority of our proposed DRL-based method in terms of higher convergence speed and reduced oscillations. Additionally, to address the reviewer’s suggestion, we have conducted a frequency domain analysis using a PID controller for comparison. This comprehensive evaluation further proves the effectiveness and innovative nature of our DRL-based approach against a strong competitor.*

*Clarification parts were highlighted on pages 15, 16 and 21.*

4. Some sentences in this article are lengthy and grammatically incorrect. Please have colleagues or professional editors who are native English speakers polish the language and standardize the reference format.

**Response:** *Thank you for your time. As said in the reply to the esteemed reviewer #1, the new form of the text got good looks and checks by professional English users. They cut out all big word flaws, long lines, and typos, too. They also fixed the look of all citations to match the journal's policies.*

*Corrections applied the entire of the paper context.*

*We hope the fixes and words will satisfy the esteemed reviewers. Once more, big thanks for your time to look at our work.*

6. PLOS authors have the option to publish the peer review history of their article ([what does this mean?](https://track.editorialmanager.com/CL0/https:%2F%2Fjournals.plos.org%2Fplosone%2Fs%2Feditorial-and-peer-review-process%23loc-peer-review-history/1/010f01994b395c21-3668d0d7-1f7d-4621-b8f9-ea83aeb4533e-000000/i8OOs0L2syv64a7ryZsKoGIL-bSCbds-LP0pfiH3V2c=228)). If published, this will include your full peer review and any attached files.

If you choose “no”, your identity will remain anonymous but your review may still be made public.

**Do you want your identity to be public for this peer review?** For information about this choice, including consent withdrawal, please see our [Privacy Policy](https://track.editorialmanager.com/CL0/https:%2F%2Fwww.plos.org%2Fprivacy-policy/1/010f01994b395c21-3668d0d7-1f7d-4621-b8f9-ea83aeb4533e-000000/Lt6hAN5qQHa3G28WGINVKvyqB3wDylUfRW4jDiS7vso=228).

Reviewer #1: No

Reviewer #2: No

Reviewer #3: No

Best regards,

Corresponding author: Jafar Keighobadi
